# Gut microbiota disorder caused by diterpenoids extracted from *Euphorbia pekinensis* aggravates intestinal mucosal damage

**DOI:** 10.1002/prp2.765

**Published:** 2021-09-14

**Authors:** Kuilong Wang, Xiaofen Xu, Aikebaier Maimaiti, Min Hao, Xianan Sang, Qiyuan Shan, Xin Wu, Gang Cao

**Affiliations:** ^1^ School of Pharmacy Zhejiang Chinese Medical University Hangzhou China

**Keywords:** diterpenoids, *Euphorbia pekinensis*, gut microbiota, intestinal mucosal, intestinal toxicity

## Abstract

Gut microbiota disorder will lead to intestinal damage. This study evaluated the influence of total diterpenoids extracted from *Euphorbia pekinensis* (TDEP) on gut microbiota and intestinal mucosal barrier after long‐term administration, and the correlations between gut microbiota and intestinal mucosal barrier were analysed by Spearman correlation analysis. Mice were randomly divided to control group, TDEP groups (4, 8, 16 mg/kg), TDEP (16 mg/kg) + antibiotic group. Two weeks after intragastric administration, inflammatory factors (TNF‐α, IL‐6, IL‐1β) and LPS in serum, short chain fatty acids (SCFAs) in feces were tested by Enzyme‐linked immunosorbent assay (ELISA) and high‐performance liquid chromatography (HPLC), respectively. The expression of tight junction (TJ) protein in colon was measured by western blotting. Furthermore, the effects of TDEP on gut microbiota community in mice have been investigated by 16SrDNA high‐throughput sequencing. The results showed TDEP significantly increased the levels of inflammatory factors in dose‐dependent manners, and decreased the expression of TJ protein and SCFAs, and the composition of gut microbiota of mice in TDEP group was significantly different from that of control group. When antibiotics were added, the diversity of gut microbiota was significantly reduced, and the colon injury was more serious. Finally, through correlation analysis, we have found nine key bacteria (Barnesiella, Muribaculaceae_unclassified, Alloprevotella, Candidatus_Arthromitus, Enterorhabdus, Alistipes, Bilophila, Mucispirillum, Ruminiclostridium) that may be related to colon injury caused by TDEP. Taken together, the disturbance of gut microbiota caused by TDEP may aggravate the colon injury, and its possible mechanism may be related to the decrease of SCFAs in feces, disrupted the expression of TJ protein in colon and increasing the contents of inflammatory factors.

## Graphical abstract

The total diterpenoids were extracted and isolated from the radix of *Euphorbia pekinensis* (TDEP), and it’s influence on gut microbiota and intestinal mucosal barrier were evaluated. Correlation analysis was conducted to find out key microbiota. In conclusion, we found the disturbance of gut microbiota caused by TDEP may aggravate the colon injury, and its possible mechanism may be related to the decrease of SCFAs in feces, disrupted the expression of TJ protein in colon and increasing the contents of inflammatory factors.

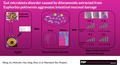

**Abbreviations**
ACET: acetate
BCA: bicinchoninic acid
BUT: butyrate
ELISA: Enzyme‐linked immunosorbent assay
EP: *Euphorbia Pekinensis*
HE: hematoxylin‐eosin.
HPLC: high‐performance liquid chromatography
ISC: intestinal stem cells
LPS: lipopolysaccharide
MMP: mitochondrial membrane potential
PROP: propionate
PVDF: polyvinylidene difluoride
SCFA: short chain fatty acids
SYNPO: synaptopodin
TBS: Tris‐buffered saline
TJ: tight junction

## 1 INTRODUCTION

*Euphorbia Pekinensis* (EP), the radix of Euphorbia pekinensis Rup, is a well‐known Chinese herb which has been used to treat gonorrhea, edema, ascites, migraines, and warts for 1000 years.^1^, ^2^ Pharmacological studies show that EP also has anti‐tumor and anti‐angiogenic activities,^3^, ^4^ and the active ingredients are some water‐soluble high polar substances. However, it is worth noting that EP can cause abdominal pain and diarrhea when used improperly.^5^, ^6^ Many studies have shown that the toxic components of EP are concentrated in the low polar parts, especially diterpenoids, which are considered to be the main toxic components. In vitro study, casbane diterpenoids from EP are toxic to many cell lines, such as LO‐2,^7^ IEC‐6^8^ and MDCK,^9^ they can induce cell apoptosis, cellular morphological change, ROS accumulation, and mitochondrial membrane potential (MMP) disruption.^7^, ^8^, ^9^ In our previous study, we demonstrated the toxic effects of casbane diterpenoids in vivo, the possible mechanism is due to the disordered expression of aquaporin in intestinal tract caused by diterpenoids from EP,^5^ and inflammation aggravates the disorder of aquaporin expression.^10^ All these studies have proved the toxic effects of diterpenoids from EP; however, the mechanism is still unclear, particularly, the existing research has not paid attention to gut microbiota, which plays an important role in the homeostasis of the gut.

Gut microbiota has been found to be related to many diseases, such as diabetes,^11^ cancer,^12^, ^13^, ^14^ chronic kidney disease,^15^, ^16^ and Parkinson's disease,^17^ the gut microbiota regulates the disease process through some key metabolites, thus, homeostasis of gut microbiota is very important to maintain the health of the body. The intestinal mucosal barrier consists of mechanical barrier, chemical barrier, biological barrier, and immune barrier,^18^ gut microbiota constitutes the biological barrier of intestinal barrier, and closely related to the integrity of intestinal immune barrier function, it shapes our immune responses throughout life. Disturbance of gut microbiota will lead to the damage of barrier function, loss of the intestinal barrier causes systemic immune activation, resulting in a wide range of extra intestinal autoimmune and inflammatory diseases.^19^ Short chain fatty acids have been found to play an important role in the relationship between gut microbiota and mucosal barrier function, they are bacterial metabolites produced in the gastrointestinal tract that are considered to be beneficial to host cell, research shows that acetate (ACET), propionate (PROP), or butyrate (BUT) may affect the intestinal stem cells (ISC) activity, differentiation, barrier function, and epithelial defense.^20^ Among these SCFAs, BUT has been the most widely studied, it shows that butyrate induces actin‐binding protein synaptopodin (SYNPO) expression in epithelial cell lines and murine colonic enteroids through mechanisms possibly involving histone deacetylase inhibition, which reveals a direct mechanistic link between microbiota‐derived butyrate and barrier restoration.^21^ All these findings suggest that gut microbiota is essential for the integrity of intestinal mucosal barrier function, and we speculated that the toxic diterpenes from *Euphorbia pekinensis* may cause severe intestinal mucosal damage by affecting gut microbiota.

In our previous study, we tested the acute toxic of total diterpenoids extracted from *Euphorbia pekinensis* (TDEP), and most studies on intestinal toxicity of *Euphorbia pekinensis* focus on acute toxicity; however, in many cases, it needs to be taken for a long time, and the toxicity under this condition is unclear. In this study, 16SrDNA sequencing was used to detect the difference of gut microbiota in mice after TDEP administration for 2 weeks, and seek for different microbiota. Histopathological section of colon and the TJ protein expression was tested to confirm the damage of intestinal mucosal barrier. The content of SCFAs in intestinal feces was also determined, and we further used antibiotic interference to verify the toxic effects of TDEP and the protective effects of SCFAs. Our findings reveal that gut microbiota disorder caused by TDEP aggravates intestinal mucosal damage.

## 2 MATERIALS AND METHODS

### 2.1 Preparation of TDEP

TDEP were extracted and isolated from the radix of EP according to previous studies.^5^ 6 known diterpenoids accounting for 85.26% of TDEP. TDEP were dissolved in methanol and detected using HPLC. Six compounds were found, accounting for 2.44%(Pekinenin G), 5.05%(Yuexiandajisu A), 9.34%((‐)‐(1S)‐15‐hydroxy‐18‐carboxycembrene), 6.67%(Pekinenin A), 57.29%(Pekinenin C), and 4.47%(Pekinenin F) of TDEP, respectively. Pekinenin C accounts for 57.29% of the total diterpenoids, it may be the main toxic component. The chemical structures of the 6 known diterpenoids of TDEP are shown in Figure S2. The HPLC chromatogram of TDEP and Mass spec profile for five known diterpenoids (1–5) are shown in Figures [Link], [Link], [Link]. Cytotoxicity data of the six diterpenoids against three gastrointestinal cell lines and fragment ions of them are shown in Tables S1 and S2.

### 2.2 Animals and treatment

Mice aged 8 weeks and weighing around 20 g were obtained from Zhejiang Chinese Medical University Laboratory Animal Research Center. They were maintained at a controlled temperature (22 ± 2℃), with a 12‐h light/dark period, and fed with standard chow for at least 1 week before any manipulations. All animal procedures were carried out in strict accordance with the Guiding Principles for the Care and Use of Laboratory Animals, as adopted by the Committee of Animal Research at Zhejiang Chinese Medical University. And the study was conducted in accordance with the Pharmacology Research & Perspectives policy for experimental and clinical studies. The animal ethics approval number for the study is SYXK (Zhe) 2018–0012.

Mice were randomly divided into five groups with equal numbers (*n* = 6): Control group, three TDEP groups (4, 8, 16 mg/kg, respectively), in antibiotic group,mice were administrated with TDEP (16 mg/kg), with 50 µg/ml clindamycin (Sigma), 50 µg/ml metronidazole (Sigma), 50 µg/ml penicillin (Sigma), 50 µg/ml neomycin (Sigma) in in sterile drinking water. The five groups were orally administrated by syringe‐feeding with distilled water (0.3 ml/kg) or TDEP. Two weeks after administration, fresh feces and colons were collected and stored in liquid nitrogen, respectively. All samples were finally stored at −80℃ for subsequent treatment.

### 2.3 Measurement of serum levels of inflammatory cytokines and lipopolysaccharide (LPS)

At the end of the treatment, all mice were sacrificed by cardiac puncture under 10% chloral hydrate (0.7 ml/100 g, i.p.). Blood was collected in dry tubes and each serum sample were stored at −80℃. The concentrations of LPS were determined using mouse LPS enzyme‐linked immunosorbent assay (ELISA) kit (MEIMIAN, 202008, Shanghai, China) according to the manufacturer's instructions. The concentrations were spectrophotometrically quantified by measuring the absorbance at 450 nm.

Levels of the inflammatory cytokines IL‐6, IL‐4, and IL‐10 were quantitatively detected using the enzyme‐linked immunosorbent assay (ELISA) kitS (MEIMIAN, MM‐0163M2, MM‐0040M2, MM‐0132M2) according to the manufacturer's protocols.

### 2.4 Western blot analysis of TJ proteins in tissue

To detect the release of TJ proteins on colon, the expression of claudin‐1, occludin, ZO‐1 were analyzed by WB. Total protein from colon was extracted with RIPA buffer, and the protein concentrations were measured via the bicinchoninic acid (BCA) assay. Then equal amounts of protein mixed with 5× bromophenol blue loading buffer and boiled for 5 min at 100℃. Proteins were separated by 10% sodium dodecyl sulfate polyacrylamide gel, followed by electroblotting onto polyvinylidene difluoride (PVDF) membrane. To prevent nonspecific binding, the membranes was blocked in 5% non‐fat milk (prepared in Tris‐buffered saline [TBS] containing 0.1% Tween‐20) for 2 h, followed incubated overnight at 4℃ with a 1:500 dilution of anti‐rabbit claudin‐1(abcam, ab15098), and 1:1000 dilutions of anti‐rabbit occludin (abcam, ab216327), anti‐rabbit ZO‐1 (abcam, ab96587), and anti‐mouse β‐actin (Boster, BM0627). Then the membrane of β‐actin was incubated with a 1:5000 dilution of horseradish peroxidase‐conjugated goat anti‐mouse IgG antibody (Boster, BA1050) for approximately 2 h, the membranes of claudin‐1, Occludin, and ZO‐1 were incubated with a 1:5000 dilution of horseradish peroxidase‐conjugated goat anti‐rabbit IgG antibody (abcam, BA1054) for approximately 2 h. After washing, protein bands were visualized using Ultra‐sensitive ECL chemiluminescence kit (Beyotime), and visualized with ChemiDoc™ Touch (Bio‐Rad).

### 2.5 Histopathological examination

The colon samples (*n* = 6) were fixed in 10% phosphate‐buffered formalin, dehydrated and then embedded with paraffin. Subsequently, the tissues were cut into 5‐μm sections and stained with hematoxylin‐eosin (HE). Representative micrographs of the colon sections were obtained using a 400× objective under a light microscope.

### 2.6 Determination of SCFAs using high performance liquid chromatography (HPLC)

Briefly, 300 mg feces that was added to 1 ml ultra‐pure water and 100 μl concentrated hydrochloric acid was fully homogenized thoroughly. Then stand for 20 min, mixed 2 times during the period. The samples were centrifuged at 4°C (13,861 × *g*, 10 min), then the supernatant was centrifuged at 4°C (866 × *g*, 5 min) after extracted by 600 μl ether for 20 min. Took 400 μl organic phase, added 500 μl 1 M NaOH and continue extraction for 20 min. Water phase was obtained after centrifuged at 4°C (866 × *g*, 5 min), and the 450 μl supernatant added to 300 μl concentrated hydrochloric acid was immediately filtered through a 0.22 μm microfiber filter. Agilent C18 column (250 × 4.6 × 5.0 mm) were used to separate SCFAs using an HPLC (e alliance 2695–2998, Waters) equipped with diode array detectors and detected at 210 nm. Mobile phase: A (acetonitrile) and B (water and 0.1% formic acid) (80% B from 0 to 5 min, 80%–75% B from 5 to 10 min, 75%–65% B from 10 to 25 min, 65%–61% B from 25 to 30 min, 61%–80% B from 30 to 35 min). The flow rate of the mobile phase was 0.8 ml/min, and the column temperature was maintained at 25℃.

### 2.7 Gut microbiota analysis

Gut microbiota DNA was extracted from each fecal sample using the E.Z.N.A.^®^Stool DNA Kit according to the manufacturer's instructions. The quality of DNA in each sample was detected by agarose gel electrophoresis, and quantified by micro nucleic acid protein analyzer (ThermoFisher). Specific primers were used to amplify the V3–V4 region of bacterial 16S rDNA via PCR [341F (5'‐CCTACGGGNGGCWGCAG‐3’) 805R (5'GACTACHVGGGTATCTAATCC‐3’)]. The PCR reactions (25 µl) were conducted using 12.5 µl Phusion Hot start flex 2X Master Mix, 5 µl specific primers, 50 ng template DNA and ddH_2_O. The PCR reactions were performed as follows: 98℃ for 40 s, followed by 35 cycles of 54℃ for 30 s, and 72℃ for 45 s, with a final extension of 72℃ for 10 min. The PCR products were verified by 2% agarose gel electrophoresis, then purified using AMPure XT beads (Beckman Coulter Genomics), quantified through Qubit (Invitrogen), and the document was obtained after evaluated on Agilent 2100 Bioanalyzer (Agilent) and Illumina (KapaBiosciences) library quantification kits. The gut microbiota profile was determined using a MiSeq high‐throughput sequencing platform (NovaSeqPE250).

### 2.8 Statistical analysis

SPSS version 16.0 for Windows (SPSS) was used for statistical analysis. Numerical data were expressed as mean ± SD. The significance of differences was examined using one‐way analysis of variance (ANOVA) procedure followed by the Dunnett's test. The correlations between microbiota and host parameters were analyzed by Spearman's correlation. Results with *p* < .05 were considered statistically significant.

## 3 RESULTS

### 3.1 Histological results

**FIGURE 1:**
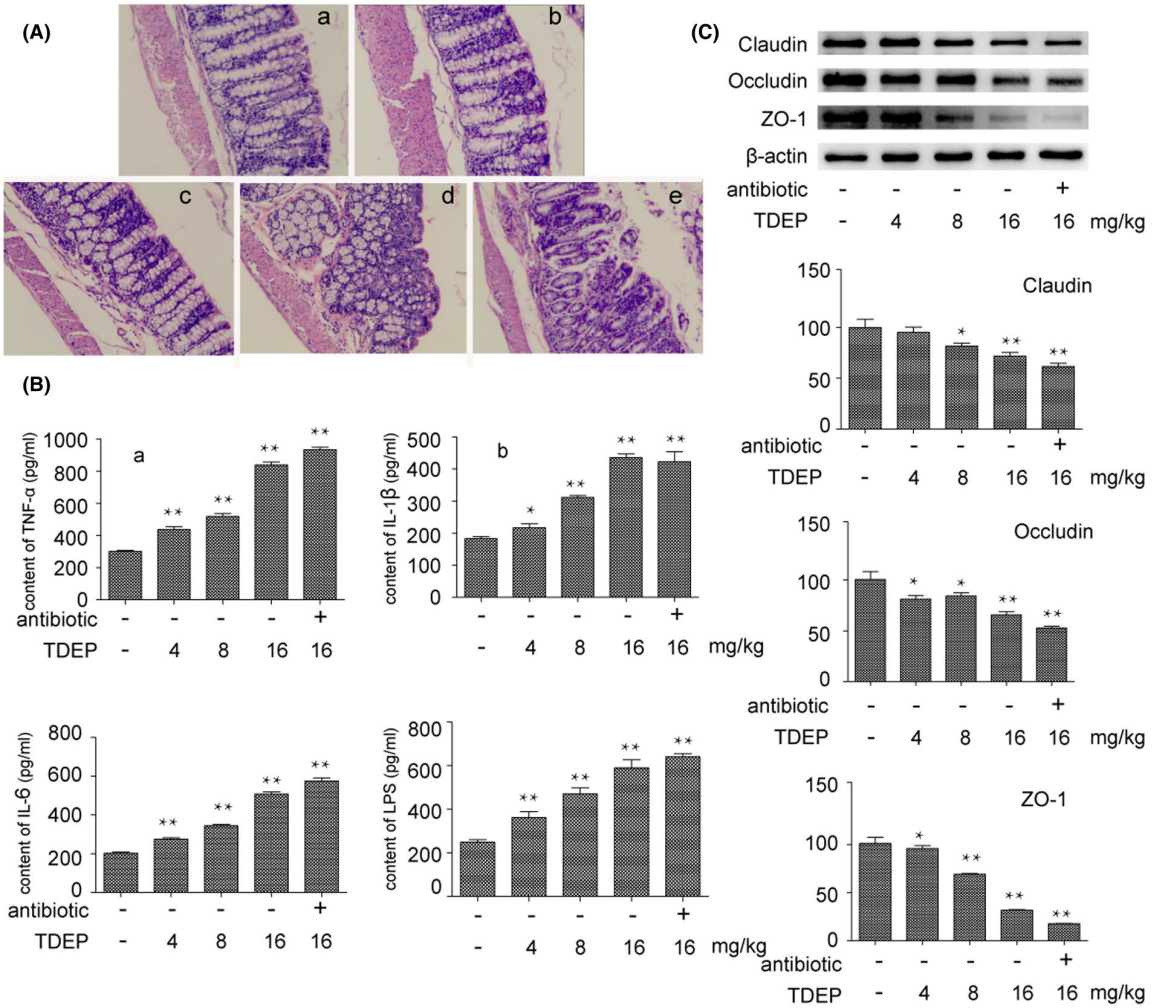
(A) Effects of different dosage of TDEP and TDEP associated with antibiotics on the histological morphology of mice colon by HE staining. (a) control group; (b) mice administered with TDEP (4 mg/kg); (c) mice administered with TDEP (8 mg/kg); (d) mice administered with TDEP (16 mg/kg); (e) mice administered with TDEP (16 mg/kg) associated with antibiotics. (B) Effects of different dosage of TDEP and TDEP associated with antibiotics on the expression of inflammatory cytokines and LPS in serum. (a) TNF‐α; (b) IL‐1β; (c) IL‐6; (d) LPS. (C) Effects of different dosage of TDEP and TDEP associated with antibiotics on the expression of TJ proteins in the mice colon. The results were normalized with β‐actin protein level, and all TJ proteins level of the control was taken as 100%. Data are represented as the mean ± SD. *p < .05, **p < .01 versus control group, respectively. *n* = 6 in each group and each assay was repeated three times

Representative HE staining of colon tissues is shown in Figure 1A. The pathological morphology of control group was normal, no inflammatory response and damage. In the high dose group of TDEP, significant mucosal damage was observed, the villi were irregular with local epithelial shedding, inflammatory infiltration of large areas of mononuclear leukocytes in the mucosa and submucosa, and there was edema between mucosal and muscular layers in the colon. In the antibiotic treatment group, mucosal damage was more serious. Inflammatory infiltration was also observed in the medium dose group, some of the epithelial cells fell off, and the edema between the colonic mucosa and muscular layer was alleviated, and the damage of low dose group was not obvious compared with control group.

### 3.2 Effects of TDEP on expression of inflammatory factors and TJ protein levels

The damage of mucosal barrier function may lead to inflammatory reaction, in this study, the expression of IL‐1β, IL‐6, and TNF‐α in serum was tested by enzyme linked immunosorbent assay (ELISA). The result shows that TDEP lead to the increase of inflammatory factors in blood in a dose‐dependent manner(*p* < .05), and the expression of inflammatory factors was even higher than TDEP high dose group in antibiotic treatment group, content of LPS in serum was also detected, the result was consistent with the inflammatory factors expression (*p* < .05).

Occludin, claudin‐1, and ZO‐1 are important tight junction proteins, they are critical for the maintenance of intestinal mucosal barrier function. The expressions of these three TJ proteins in colon were detected, as shown in Figure 1C, after TDEP administration, the expression of TJ protein in colon of mice was significantly decreased, and the expression of TJ protein in the colon of antibiotic treatment group was lower than that of high dose group (*p* < .05).

### 3.3 The contents of SCFAs in feces

**FIGURE 2:**
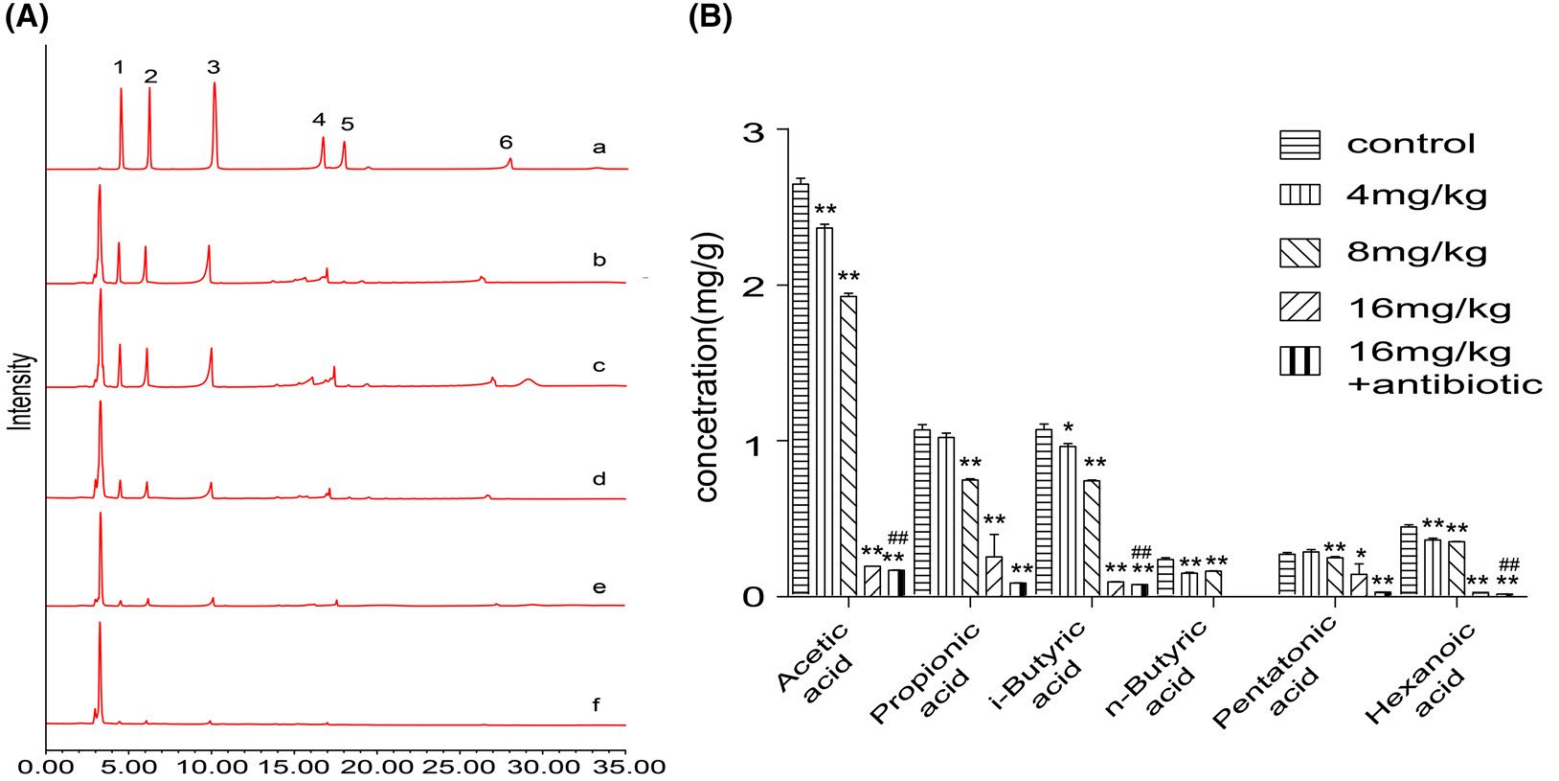
The contents of the SCFAs in the feces. (A) HPLC chromatogram of SCFAs standard and different samples: (a) standard. (1) acetic acid, (2) propionic acid, (3) i‐butyric acid, (4) n‐butyric acid, (5) pentatonic acid, (6) hexanoic acid; (b) control group; (c) mice administered with TDEP (4 mg/kg); (d) mice administered with TDEP (8 mg/kg); (e) mice administered with TDEP (16 mg/kg); (f) mice administered with TDEP (16 mg/kg) associated with antibiotics. (B) Effects of different dose of TDEP on the contents of the SCFAs. Data are represented as the mean ± SD. *p < .05, **p < .01 versus control group, respectively; ^#^ *p* < .05, ^##^ *p* < .01 versus TDEP (16 mg/kg). *n* = 6 in each group and each assay was repeated three times

The contents of SCFAs in mice feces at different doses of TDEP were tested. As shown in Figure 2, acetic acid, propionic acid, and butyric acid are the main SCFAs in feces, accounting for about 80% of SCFAs. In low dose of TDEP(4 mg/kg), there was significant difference in the contents of acetic acid, i‐butyric acid, n‐butyric acid, and hexanoic acid (*p* < .05). In medium dose group, all the SCFAs were decreased (*p* < .05). While in high dose group, all SCFAs were significantly decreased (*p* < .05). Compared with high dose group, acetic acid, i‐butyric acid, and hexanoic acid were significantly decreased in antibiotic group (*p* < .05), and n‐butyric acid was not detected in high‐dose and antibiotic groups.

### 3.4 16SrDNA sequencing

These sequence data have been submitted to the Sequence Read Archive(SRA) databases under accession number SUB8556324.

### 3.5 Alpha and Beta diversity analysis

**FIGURE 3:**
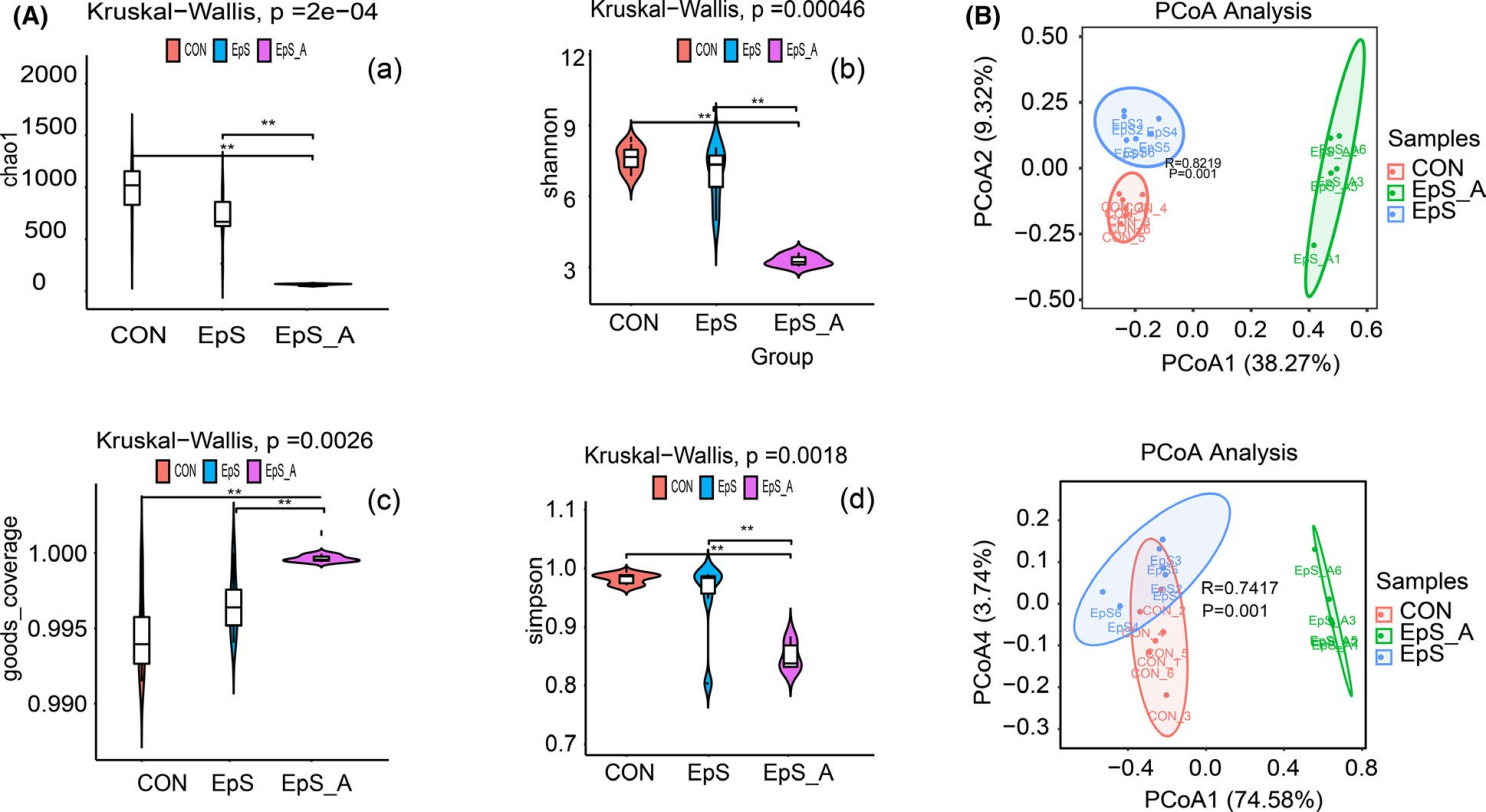
Alpha and Beta diversity analysis. (A) Alpha diversity analysis of species distribution: (a) Chao1; (b) Shannon; (c) Goods_coverage; (d) Simpson. The data showed that there was significant difference between the antibiotic group and the other two groups, but there was no significant difference between TDEP group and control group. (B) Weighted and unweighted PCoA analysis. The PCoA analysis showed a clear separation of the TDEP group from the control group and the antibiotic treatment group

Alpha diversity analysis is used to evaluate the species diversity of different treated groups, which includes the Chao1, Observed species, Goods_coverage, Shannon, and Simpson indexes. In this research, Chao 1 index and Goods_coverage index results showed that species value was significantly different when antibiotics were used. However, there was no significant difference between control group and TDEP group. Community diversity was estimated using the Shannon index and the Simpson index, the results are shown in Figure 3A b and d , compared with control and TDEP group, Shannon index of antibiotics‐treated group was lower, and Simpson index was closer to 0, which means there were few species in the antibiotics treated group, and the values between control and TDEP group were also not obvious. Therefore, the diversity of control and TDEP group was not affected, antibiotics‐treated group could obviously affect the diversity.

Beta diversity refers to the species diversity among different environmental communities. In this research, we used weighted and unweighted principal coordinates analysis (PCoA) to compare the community composition differences between different samples. The results showed that TDEP group changed the gut microbiota significantly from control group, and antibiotic treatment group showed an obvious separation of other two groups. These indicated that there were significant differences in gut microflora among the three groups (Figure 3B).

### 3.6 The microbial community structures at the phylum and genus levels

**FIGURE 4:**
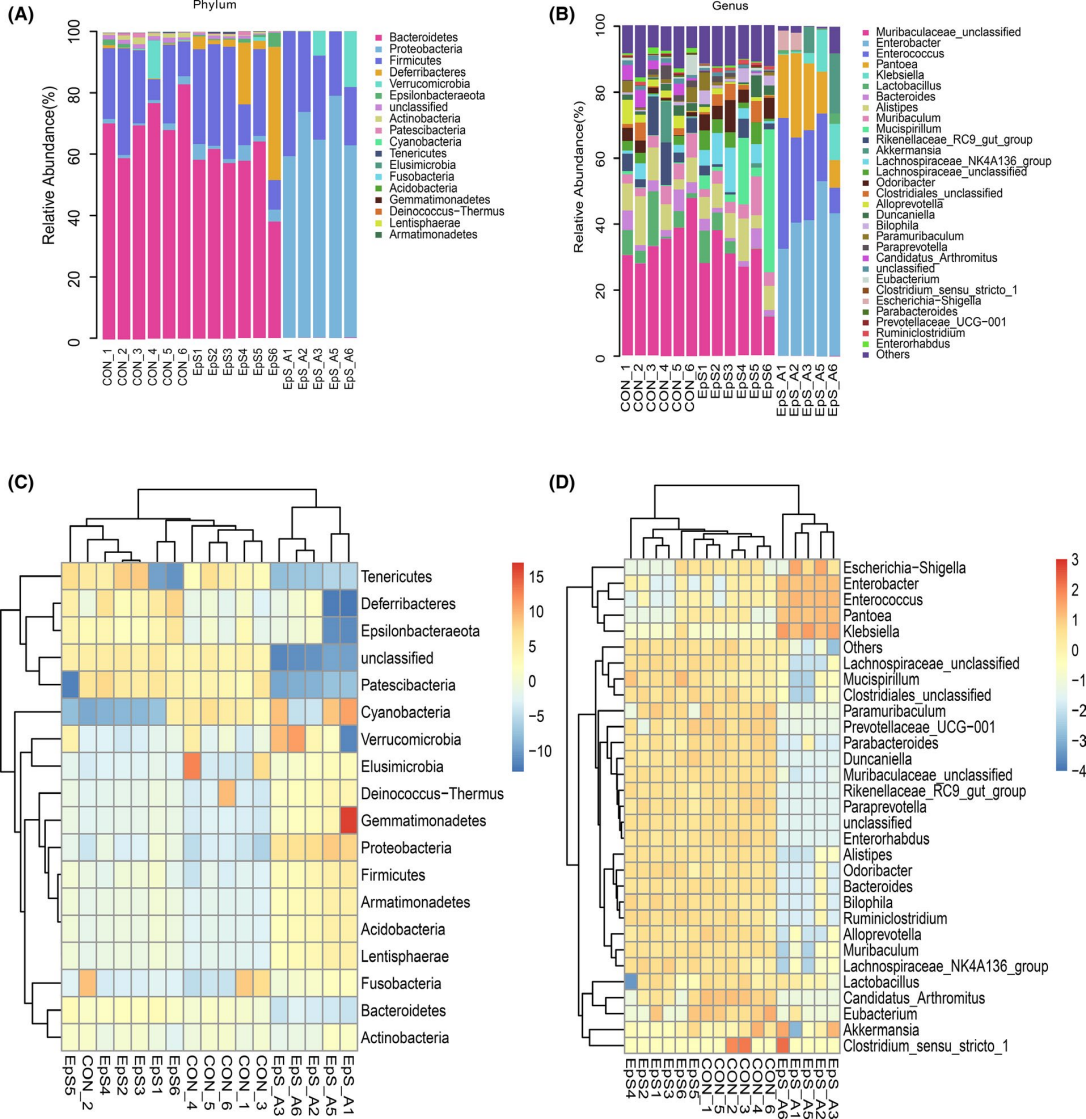
Differences of microbial community structures at the phylum and genus levels. (A) Column of microbial at phylum level in each group; (B) Column of microbial at genus level in each group; (C) Heatmap and the cluster analysis of the top 18 abundance bacterial phyla; (D) Heatmap and the cluster analysis of the top 30 abundance bacterial genera

From the diversity results, we can see that the species composition was quite different between three groups. We further selected the highest abundance from the phylum and genus level to analyze the species differences among three groups. As illustrated by Figure 4A, at the phylum level, Bacteroidetes, Firmicutes, Actinobacteria, and Patescibacteria were the main phyla of control group and TDEP group, although the species are similar, their composition is different. However, antibiotic‐treated group was quite different, in this group, Proteobacteria (70.63%) and Firmicutes (22.96%) became the main phyla, the abundances of other phyla were lower than that in other two groups. The abundance of Proteobacteria (3.04%) in TDEP group was higher than that in control group (1.49%) (*p* < .05), although the abundances of Deferribacteres, Firmicutes, Epsilonbacteraeota, and Tenericutes also increased, there were no significant difference. At the same time, the abundances of Bacteroidetes (56.23%), Actinobacteria (0.34%), Cyanobacteria (0.00%) in TDEP group were lower than that in control group (71.16%, 1.31%, 0.03%) (*p* < .05). The abundances of other phyla did not change significantly.

At the Genus level, we selected the top 30 species for assessment, and the results showed that the distribution of the gut microbiota was significantly altered among the three groups. Compared with control group, the relative abundances of Enterobactacter, Alloprevotella, Alloprevotella, Rikenellaceae_RC9_gut_group, Parabacteroides, and Enterorhabdus were siganificantly downregulated by TDEP administration. The relative abundances of Lachnospiraceae_NK4A136_group, Lachnospiraceae_unclassified, Bilophila, Mucispirillum, and Ruminiclostridium were increased significantly. At the same time, we observed that the abundances of antibiotic treatment group were quite different, Enterobacter and Enterococcus became the main species, accounting for 66.26%, but in control group and TDEP group, the numbers were 0.07% and 0.03%, respectively. Meanwhile, some bacteria with low abundances in the control group and TDEP group increased in the antibiotic group, such as Pantoea (17.19%), Klebsiella (5.50%), and Escherichia‐Shigella (2.25%), and many microbiota species cannot even be detected. In the cluster analysis of the bacterial phyla and genera, it was found that the distributions of these phyla and genera in the TDEP group were closer to that in the control group than in antibiotic group.

### 3.7 LEfSe analysis in TDEP and antibiotic‐treated group

**FIGURE 5:**
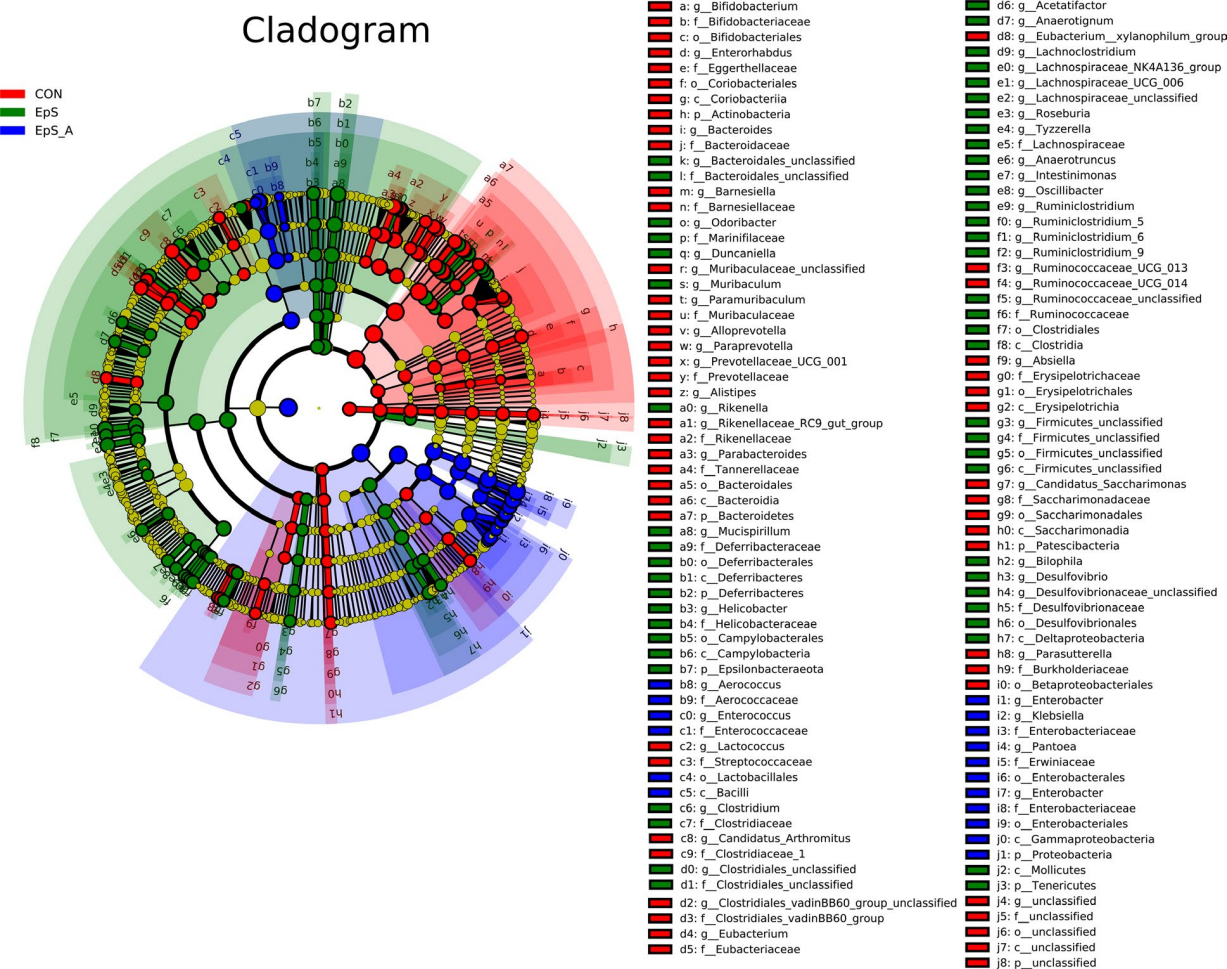
Specific biomarkers of TDEP and antibiotic treated group

To define which bacterium might be responsible for colon injury induced by TDEP, linear discriminant analysis (LDA) effect size (LEfSe) was used to analyze the differences among the three groups from the phylum level to the genus level, and the magnitudes of effects of the different species biomarkers were assessed by LDA (Figure S1). The results showed that gut microbiota differed significantly among three group, about 59 biomarkers were found (Figure 5). We removed the bacteria with relative abundance less than 0.1% for further analysis, and 35 bacterial genera were selected. The correlations between the 35 bacterial genera and biochemical parameters were analyzed by Spearman's correlation analysis, eventually, 9 bacteria genera with significant correlation with some biochemical parameters are shown in Table 1. Notably, in antibiotic‐treated group, some opportunistic pathogens such as Klebsiella were detected, and this may be one of the reasons why it is more toxic.

**TABLE 1:** Correlation between different bacteria genus and biochemical parameters by Spearman correlation analysis

		Barnesiella	Muribaculaceae_unclassified	Alloprevotella	Candidatus_Arthromitus	Enterorhabdus	Alistipes	Bilophila	Mucispirillum	Ruminiclostridium
IL‐1	*r*	−.**639**	−.**594**	−.392	−.**722**	−.**895**	−.457	.**606**	.**855**	.**664**
	*p*	.**025**	.**042**	.208	.**008**	.**000**	.065	.**037**	.**000**	.**018**
IL‐6	*r*	−.**761**	−.531	−.462	−.**704**	−.**867**	−.**710**	.**648**	.**886**	.748
	*p*	.**004**	.075	.131	.**011**	.**000**	.**000**	.**023**	.**000**	.005
TNF‐α	*r*	−.**691**	−.434	−.573	−.**781**	−.**832**	−.**757**	.**697**	.**904**	.559
	*p*	.**013**	.159	.051	.**003**	.**001**	.**000**	.**012**	.**000**	.059
LPS	*r*	−.**745**	−.**760**	−.469	−.**779**	−.**893**	−.401	.**796**	.**932**	.**687**
	*p*	.**005**	.**004**	.124	.**003**	.**000**	.111	.**002**	.**000**	.**014**
ZO‐1	*r*	.**773**	.226	.495	.**708**	.**610**	.**796**	−.503	−.**666**	−.**717**
	*p*	.**003**	.480	.102	.**010**	.**035**	.**000**	.096	.**009**	.**009**
Occludin	*r*	.**745**	.141	.**636**	.**743**	.537	.**651**	−.368	−.**595**	−.537
	*p*	.**005**	.661	.**026**	.**006**	.072	.**005**	.239	.**021**	.072
Claudin‐1	*r*	.**773**	.226	.495	.**718**	.574	.**638**	−.503	−.**666**	−.**638**
	*p*	.**003**	.480	.102	.**009**	.051	.**006**	.096	.**018**	.**026**
Acetic acid	*r*	.**705**	.**650**	.**608**	.**719**	.**881**	.**730**	−.**602**	−.**883**	−.**580**
	*p*	.**010**	.**022**	.**036**	.**009**	.**000**	.**001**	.**038**	.**000**	.**048**
Propionic acid	*r*	.**821**	.559	.350	.**613**	.**622**	.**613**	−.560	−.**701**	−.**650**
	*p*	.**001**	.059	.265	.**034**	.**031**	.**009**	.058	.**011**	.**022**
i‐Butyric acid	*r*	.**705**	.462	.**713**	.**802**	.**587**	.**673**	−.504	−.**662**	−.406
	*p*	.**010**	.131	.**009**	.**002**	.**045**	.**003**	.094	.**019**	.191
n‐Butyric acid	*r*	.**740**	.552	.411	.**766**	.**896**	.**514**	−.**692**	−.**912**	−.**664**
	*p*	.**006**	.063	.185	.**004**	.**000**	.**035**	.**013**	.**000**	.**018**
Pantatonic acid	*r*	.305	.**587**	.455	.347	.238	.**534**	−.476	−.354	−.021
	*p*	.335	.**045**	.138	.268	.457	.**027**	.117	.259	.948
Valeric acid	*r*	.**789**	.385	.420	.**689**	.**578**	.**666**	−.537	−.**646**	−.560
	*p*	.**002**	.216	.174	.**013**	.**049**	.**004**	.072	.**023**	.058

Significant correlations (*p* < .05) are in bold.

### 3.8 **Effects of TDEP on microbial community functions predicted by PICRUSt**.

**FIGURE 6:**
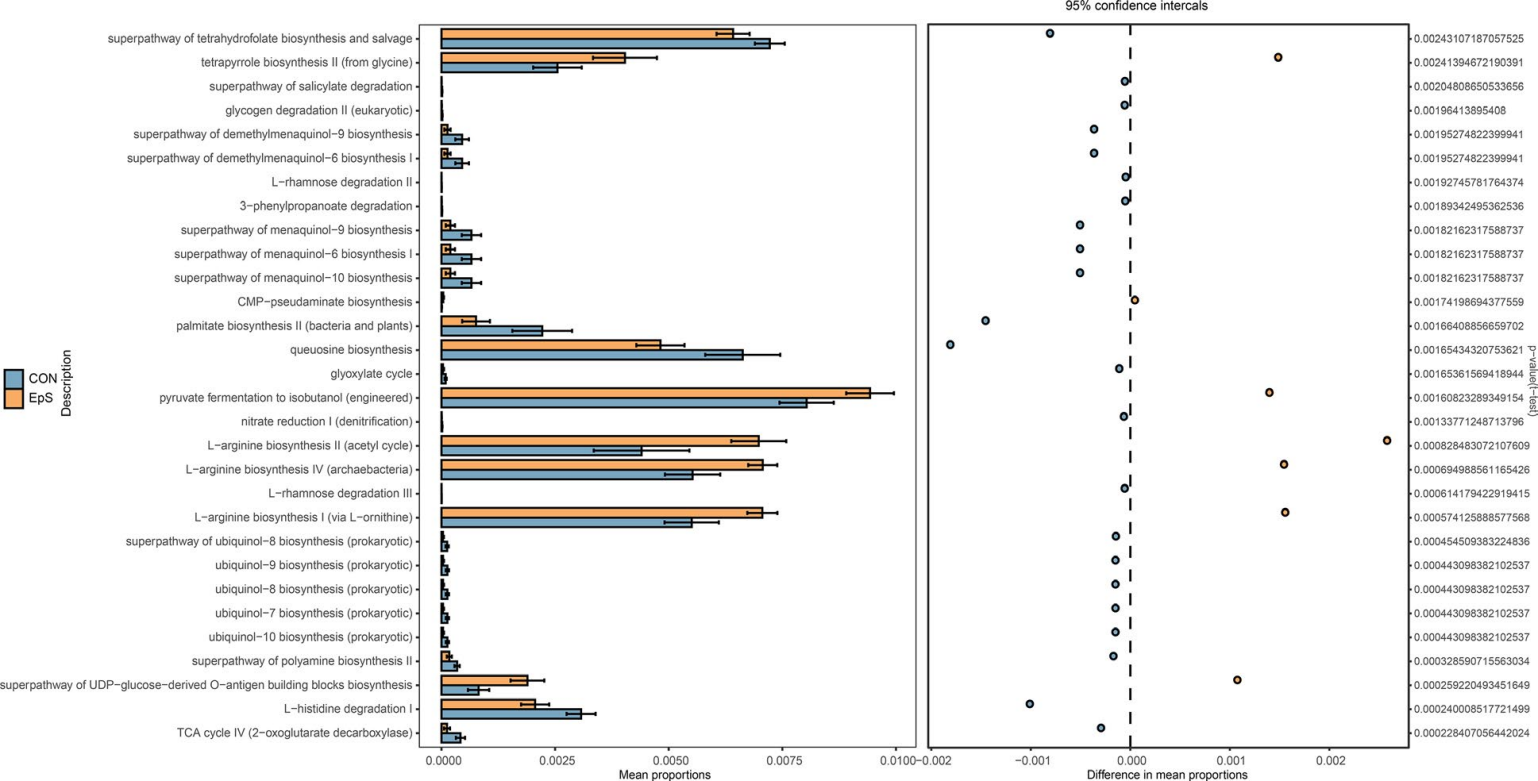
Effects of TDEP on microbial community functions predicted by PICRUSt

As shown in Figure 6, seven functional modules were significantly enriched in TEDP group, such as tetrapyrrole biosynthesis II (from glycine), CMP‐pseudaminate biosynthesis, pyruvate fermentation to isobutanol (engineered), L‐arginine biosynthesis II (acetyl cycle), L‐arginine biosynthesis IV (archaebacteria), L‐arginine biosynthesis I (via L‐ornithine), and superpathway of UDP‐glucose‐derived O‐antigen building blocks biosynthesis. Twenty‐three functional modules were depleted. The intervention of TDEP contributed to the functional difference of gut microbiota.

### 3.9 Correlation between the abundances of different bacterial genera and biochemical parameters

As shown in Table 1, ten bacterial genera with significant changes in abundance after TDEP administration were selected, and the relationships between them with inflammatory factors, TJ proteins, SCFAs were assessed. The results showed Barnesiella, Muribaculaceae_unclassified, Candidatus_Arthromitus and Enterorhabdus were negatively correlated with inflammatory factors, while Mucispirillum, Bilophila, and Ruminiclostridium were positively correlated with the expression of one or more inflammatory factors. At the same time, Barnesiella, Alloprevotella, Candidatus_Arthromitus, and Enterorhabdus were significantly positively correlated with the TJ proteins expression, such as claudin‐1, occludin, and ZO‐1, while Mucispirillum and Ruminiclostridium were significantly negatively correlated with the expression of TJ proteins. The levels of SCFAs were positively correlated with Barnesiella, Muribaculaceae_unclassified, Alloprevotella, Candidatus_Arthromitus and Enterorhabdus, and negatively correlated with Mucispirillum, Bilophila, and Ruminiclostridium.

## 4 DISCUSSION

In our previous study, we have proved the acute toxicity of TDEP, and possible mechanism is related to the induction of intestinal inflammatory response and interference with aquaporins. Previous studies have confirmed that diterpenes from *Euphorbia pekinensis* can induce apoptosis on the intestinal cells. However, there was few researches paid attention to the long‐term toxicity of *Euphorbia pekinensis* so far. In this research, we designed a two‐week oral administration of TDEP for mice, and the intestinal tissue damage was identified by pathological section. Different from acute toxicity test, serious colon tissue injury was observed in middle dose and high dose groups while there was no obvious injury of colon tissue in acute toxicity test. From the results of pathological morphology, goblet cells were completely damaged in the high dose group, and large amount of immune cells infiltration were observed, which would produce cytokines and chemokines that further amplifying local inflammation.

Studies have shown that inflammation is closely related to the expression of TJ protein, and they are essential for the maintenance of intestinal barrier function. Pioglitazone ameliorates DSS‐induced colitis and attenuates colitis‐associated mechanical hyperalgesia, with improving integrity of the intestinal mucosal barrier by directly upregulating tight junction protein ZO‐1 through the PPARγ‐tight junction protein signaling.^22^ And CS may reduce the expression of TNF‐α, promote the expressions of TJ proteins such as claudin‐1, occludin, and ZO‐1 to maintain the intestinal mucosal barrier function for attenuating UC in mice.^23^ According to the above results, this study further detected the TJ proteins in colon and inflammatory factors in serum. It has been pointed out that TJ proteins such as ZO‐1, occludin, and claudin‐1 are very important to maintain the function of intestinal mucosal mechanical barrier. In this study, the results showed that expression of tight junction proteins were decreased by TDEP administration in dose‐dependent manner, suggesting that intestinal mucosal barrier was seriously damaged, and harmful substances were more easily to enter the blood. The conjecture was verified in further experiments, as shown in Figure 1B, the level of LPS in serum of high dose group was nearly three times that of control group. As we all known, LPS is product of Gram‐negative bacteria, which induce inflammatory reaction through Toll‐like receptor, leading to intestinal dysfunction and even other organ damage.^24^ We also found that the expression of inflammatory factors also showed a dose‐dependent relationship, all these results were consistent with the histopathological damage. However, the mechanism of TDEP induced colon injury remained unclear.

Gut microbiota has been shown to be associated with tight junction proteins expression and is essential for maintaining intestinal physicochemical barrier.^25^ To investigate whether gut microbiota played a role in TDEP induced intestinal toxicity, antibiotics were added to interfere with the gut microbiota when TEDP was given to mice. It was worth noting that the damage of colon tissue was more obvious when antibiotics were added, the level of inflammatory factors in serum increased, and the expression of tight junction proteins was lower. These indicated that when the abundance of some bacteria was inhibited by antibiotics, the protective effect of bacteria on intestinal mucosa was also reduced, which eventually lead to the aggravation of colon injury.

So far, our study showed that TDEP could lead to the damage of intestinal mucosal barrier, and inhibition of gut microbiota would aggravate the damage of colon, but the correlation between them was not clear. Some studies have shown that gut microbiota can protect intestinal barrier by secreting SCFAs.^26^, ^27^ SCFAs are products of gut microbiota and play considerable roles in colonic health and integrity. SCFAs mainly consistent of acetate, butyrate, and propionate, which may affect the expression of TJ proteins and inflammatory factors, and playing an important role in promoting epithelial barrier function and wound healing.^21^, ^28^ Therefore, we speculated that TDEP may also affect the expression of SCFAs in feces. We detected the SCFAs by HPLC, and found that the content of all the SCFAs was decreased by TDEP administration in a dose‐dependent manner. And the decrease of antibiotic group was more significant, some SCFAs in feces such as n‐butyric acid had not even been detected. Combined with the previous experimental results, we found that the lower the contents of SCFAs in feces, the more serious colon tissue damage occurred. These results suggested that SCFAs secreted by gut microbiota might play a role in TDEP induced colon injury.

To find out the potential microbiota which associated with biochemical parameters closely related to colon injury, 16SrDNA high‐throughput sequencing technology was used to study the changes of gut microbiota after TDEP intervention. The results showed there were gut microbiota disorders in TDEP group. Although there was no significant change in alpha diversity of gut microbiota after intragastric administration of TDEP, there were significant differences in the gut microbiota abundance between TDEP groups and control group. PCoA analysis showed that the three groups could be significantly separated. In the species analysis, we first analyzed the difference of microbiota at phylum level, Proteobacteria was increased, while Bacteroidetes, Actinobacteria, Cyanobacteria were decreased in TDEP group, other changes were not significant. However, in antibiotic group, only Proteobacteria, Firmicutes, Verrucomicrobia, and Bacteroidetes could be detected, which was consistent with the report,^29^ the decrease in gut microbiota biodiversity may be the cause of the most serious injury in the antibiotic group.

Through species analysis and LEfSe analysis, we had selected 59 bacteria genera that contributed to colon injury. To find the most valuable bacteria genus, we combined these bacteria genera with biochemical parameters by Spearman correlation analysis, and the correlation coefficient and significant difference value of bacteria genus related to biological parameters were recorded. Finally, we screened out nine bacteria genera, five decreased bacteria genera such as Barnesiella, Muribaculaceae_unclassified, Alloprevotella, Candidatus_Arthromitus and Enterorhabdus were negatively correlated with inflammatory factors and positively correlated with TJ proteins and SCFAs, which meant they had a protective effect on the colon. Interestingly, they were decreased after TDEP administration, and among them, Barnesiella, Candidatus_Arthromitus, Alistipes and Enterorhabdus had the best correlation with biochemical parameters, and the beneficial effects of these three species on the intestinal barrier had been confirmed in previous studies. Yang, et al.^30^ found that increased abundance of Barnesiella in gut micraobiota might be closely associated with downregulation of NF‐κB and inhibition of TNF‐α activation, which eventually lead to the relief of enteritis symptoms in mice with DSS‐induced colitis. Furthermore, it was reported that Barnesiella might be related to the secretion of SCFAs.^31^ Alistipes is a SCFAs‐producing bacterium, which has protective effects against some diseases, including liver fibrosis, colitis, cancer immunotherapy, and cardiovascular disease.^32^ The role of Enterorhabdus is not very clear, in a study of the effects of smoking on Crohn's disease, the relative abundance of the genera Collinsella, Enterorhabdus, and Gordonibacter in smoking patients with Crohn's disease was reduced compared with nonsmoking patients.^33^ In another study, GFP‐Cr could significantly increase the relative abundance of Enterorhabdus in diabetes mellitus mice.^34^ These findings suggested that Enterorhabdus may play a positive role in maintaining intestinal barrier function. Candidatus Arthromitus ^35^ was proved closely related to the intestinal mucosal immunity of the host, which promoted immune maturation and enhances host resistance, and it was difficult to recovery after large doses of antibiotics treatment. All these bacteria genera were not detected in antibiotic group, which may be one of the reasons for the strong toxicity of the antibiotic group.

On the other hand, the abundances of Mucispirillum, Bilophila, Ruminiclostridium increased after TEDP adminstration. Zhang, et al.^36^ found that Shen‐Ling‐ Bai‐Zhu‐San could improve functional dyspepsia by reducing functional dyspepsia biomarkers including Prevotella, Mucispirillum, and Akkermansia. Bilophila wadsworthia, which belong to the Bilophila genera, had been proved to promote higher inflammation, intestinal barrier dysfunction and bile acid dysmetabolism, leading to higher glucose dysmetabolism and hepatic steatosis,^37^ and it had also been confirmed to be associated with colorectal cancer.^38^ The increase of these pathogens is another cause of colon injury caused by TDEP. What's more, Klebsiella and Escherichia‐Shigella were found in antibiotic group, both of them were pathogenic bacteria, but they were not detected in other two groups. We speculated that antibiotics inhibit the original gut microbiota, which made these pathogens colonize more easily and caused serious damage.

Moreover significant different functional profiles between different groups were predicted by PICRUSt. As the results showed, decrease of glyoxylate cycle and TCA cycle were observed in TDEP group, the glyoxylate cycle was a variation of the TCA cycle, and they were the hub for energy metabolism, the decrease of them meant that the energy metabolism of mice was reduced. Biosynthesis of ubiquinol 7−10 was also significantly reduced, ubiquinol also called coenzyme Q, was a well‐known antioxidant molecule, the reduction of them was not conducive to the development of antioxidant defenses in colon. There were also some metadata pathways changed after TDEP administration. However, whether they are the cause of TDEP induced colon injury need further study.

## 5 CONCLUSIONS

The experiment confirmed for the first time that colon injury induced by TDEP is associated with disturbance of gut microbiota. Through the determination of inflammatory factors in serum, tight junction proteins in colon and short chain fatty acids in feces, the damage of TDEP to colon was confirmed. The colon injury was more obvious when antibiotics were added, which suggested that some gut bacteria might play an active role in TDEP‐induced colon injury. Through the correlation analysis, 7 bacteria that are beneficial to colonic function were identified (Barnesiella, Muribaculaceae_unclassified, Alloprevotella, Candidatus_Arthromitus, Enterorhabdus, Alistipes), and 3 bacteria that were harmful to colonic function were found (Bilophila, Mucispirillum, Ruminiclostridium). This study reminded us that attention should be paid to the changes of gut microbiota when using traditional Chinese medicine for a long time to avoid adverse effects. The regulation of these bacteria can improve intestinal diseases such as inflammatory bowel disease.

## DISCLOSURE

Authors have no conflict of interest to declare.

## AUTHOR CONTRIBUTIONS

KL Wang designed and performed experiments, writing, and editing of the manuscript. XF Xu contributed to experiments, writing, and editing of the manuscript. A Maimaiti, M Hao, XN Sang, QY Shan, and X Wu were responsible for the collection, analysis, and interpretation of animal data and human data. C Gang directed this study, and wrote, and edited the manuscript. All authors have approved the submitted manuscript.

## Supporting information

Fig S1

Fig S2

Fig S3

Fig S4

Fig S5

Table S1

Table S2

## Data Availability

The data that support the findings of this study are opened available in SRA (SUB8556324).
