# Supplementary figures and images for: Gut microbiota disorder caused by diterpenoids extracted from Euphorbia pekinensis aggravates intestinal mucosal damage
Source: Pharmacol Res Perspect. 2021 Sep 14;9(5):e00765. doi: 10.1002/prp2.765 (PMC8440943; doi:10.1002/prp2.765)

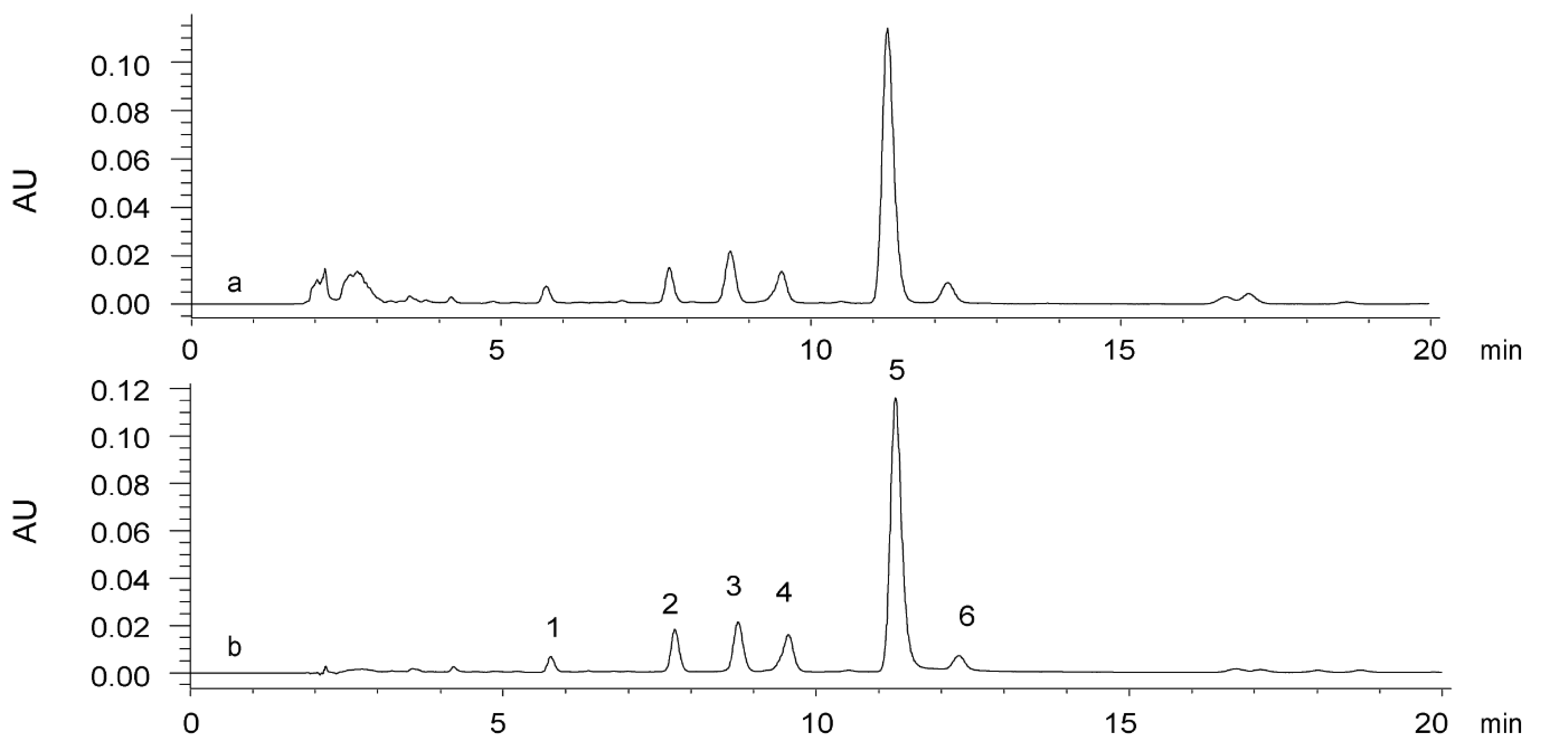

Supplement: Supplementary file 3 — Fig S3 [file PRP2-9-e00765-s005.tif]

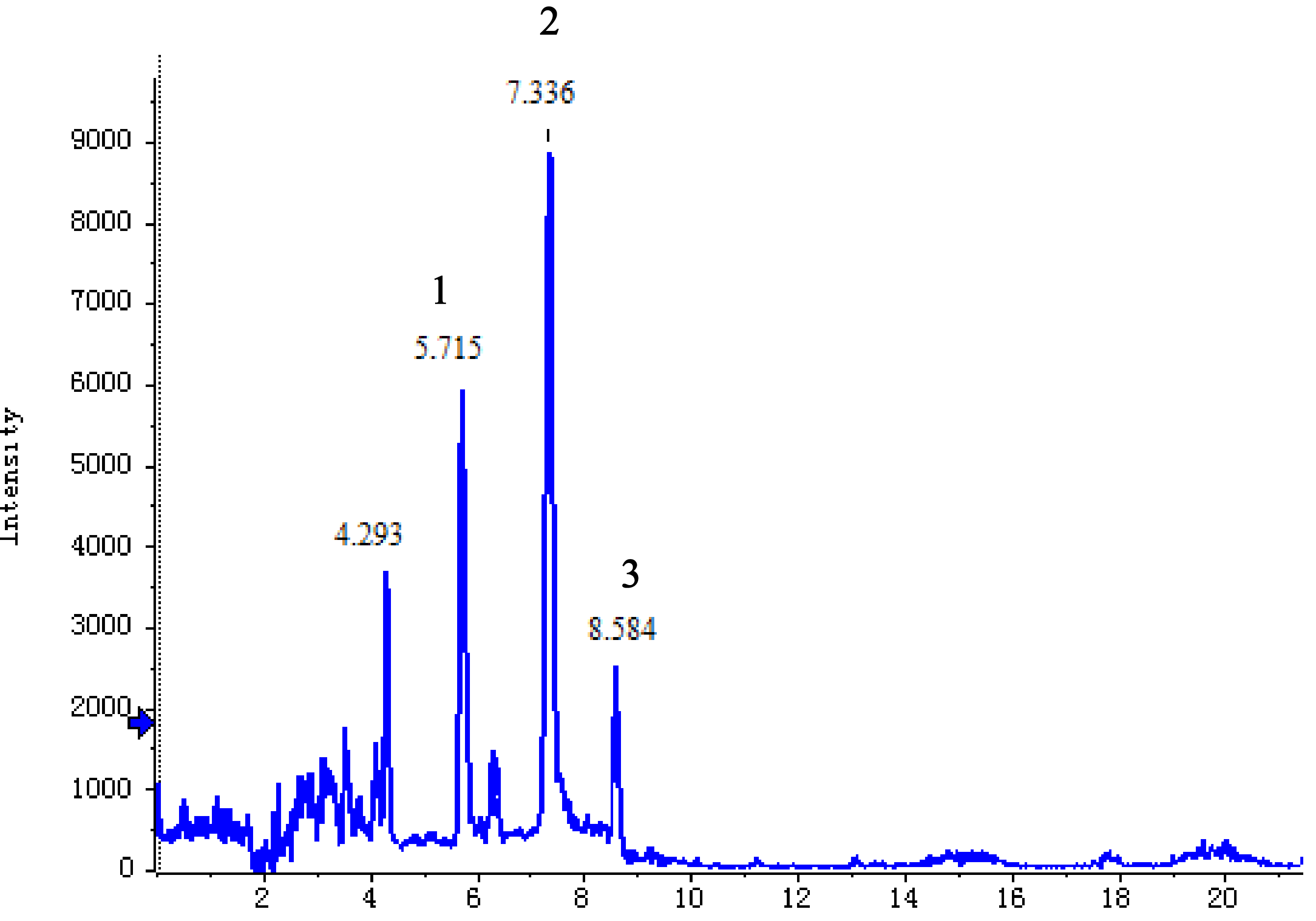

Supplement: Supplementary file 4 — Fig S4 [file PRP2-9-e00765-s003.tif]

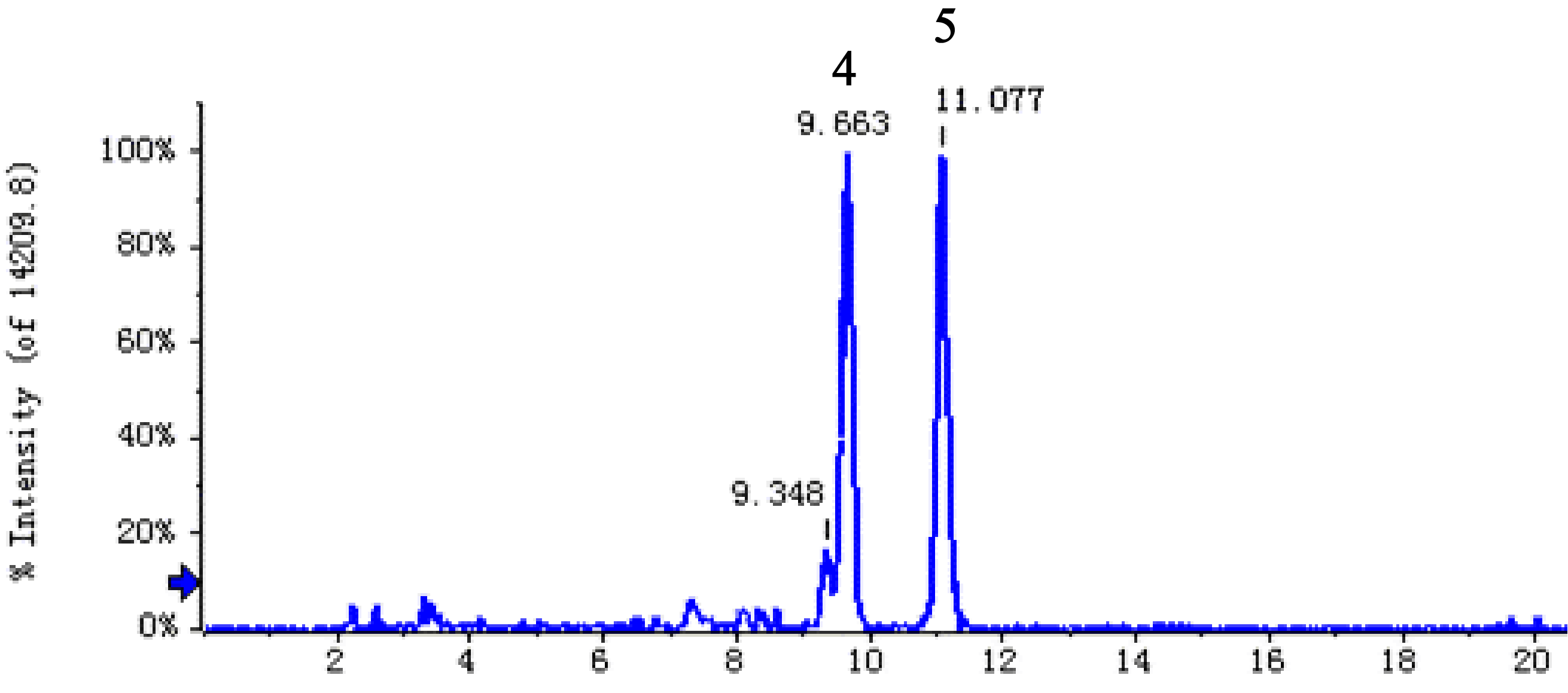

Supplement: Supplementary file 5 — Fig S5 [file PRP2-9-e00765-s002.tif]
