# Supplementary material for: Gut microbiota disorder caused by diterpenoids extracted from Euphorbia pekinensis aggravates intestinal mucosal damage
Source: Pharmacol Res Perspect. 2021 Sep 14;9(5):e00765. doi: 10.1002/prp2.765 (PMC8440943; doi:10.1002/prp2.765)
Supplement: Supplementary file 6 — Table S1 [file PRP2-9-e00765-s007.docx]

Table S1. Cytotoxicity data for compounds against three gastrointestinal cell lines.

| Compound | | IC50(μmol/L) | | |
| --- | --- | --- | --- | --- |
|  |  | BGC823 | IEC-6 | HT-29 |
| 1 | Pekinenin G | 42.7 | 39.6 | 47.8 |
| 2 | Yuexiandajisu A | 15.6 | 49.6 | 25.1 |
| 3 | (-)-(1S)-15-hydroxy-18-carboxycembrene | 54.8 | 85.5 | 110.7 |
| 4 | Pekinenin A | 25.1 | 46.3 | 30.2 |
| 5 | Pekinenin C | 12.1 | 31.6 | 11.3 |
| 6 | Pekinenin F | 53.9 | 87.2 | 70.1 |
