# Supplementary material for: Gut microbiota disorder caused by diterpenoids extracted from Euphorbia pekinensis aggravates intestinal mucosal damage
Source: Pharmacol Res Perspect. 2021 Sep 14;9(5):e00765. doi: 10.1002/prp2.765 (PMC8440943; doi:10.1002/prp2.765)
Supplement: Supplementary file 7 — Table S2 [file PRP2-9-e00765-s004.docx]

| T_R_/min | [M-H]^-^ | Fragment ions | Formula | Mass Error  (ppm) | Purity Score |
| --- | --- | --- | --- | --- | --- |
| 4.293, 5.715,  7.336, 8.584 | 317 | 317,273,180, 136,112,68 | C_20_H_30_O_3_ | -2.6 | 94.7% |
| 9.663, 11.077 | 301 | 301,164,120, 81 | C_20_H_30_O_2_ | -2.6 | 87% |

Table S2. Identification of diterpenoids from *Euphorbia Pekinensis*
